# Supplementary material for: Dihydroartemisinin-Loaded Magnetic Nanoparticles for Enhanced Chemodynamic Therapy
Source: Front Pharmacol. 2020 Mar 10;11:226. doi: 10.3389/fphar.2020.00226 (PMC7076125; doi:10.3389/fphar.2020.00226)
Supplement: Supplementary file 1 [file Data_Sheet_1.docx]

Supplementary Material

Dihydroartemisinin-loaded Magnetic Nanoparticles for Enhanced Chemodynamic Therapy

**Shengdi Guo^1^, Xianxian Yao^1^, Qin Jiang^1^, Kuang Wang^1^, Yuanying Zhang^1^, Haibao Peng^2*^, Jing Tang^3*^ and Wuli Yang^1*^**

^1^ State Key Laboratory of Molecular Engineering of Polymers, Department of Macromolecular Science, Fudan University, Shanghai, China

^2^ Department of Pharmaceutical Sciences, Shanghai University of Traditional Chinese Medicine, Shanghai, China

3 Department of Materials Science and Engineering, Stanford University, Stanford, California 94305, USA.

*** Correspondence:**W. L. Yang
[wlyang@fudan.edu.cn](mailto:wlyang@fudan.edu.cn)
J. Tang
jingtang@stanford.edu
H. B. Peng
haibaopeng@gmail.com

# Supplementary Table S1. The DLS data of MNP and drug-loaded MNP.

| **Samples** | **Size (nm)** | **PDI** |
| --- | --- | --- |
| **MNP** | 200 | 0.013 |
| **MNP-ART** | 212 | 0.065 |
| **MNP-DHA** | 204 | 0.026 |
| **MNP-AS** | 204 | 0.092 |


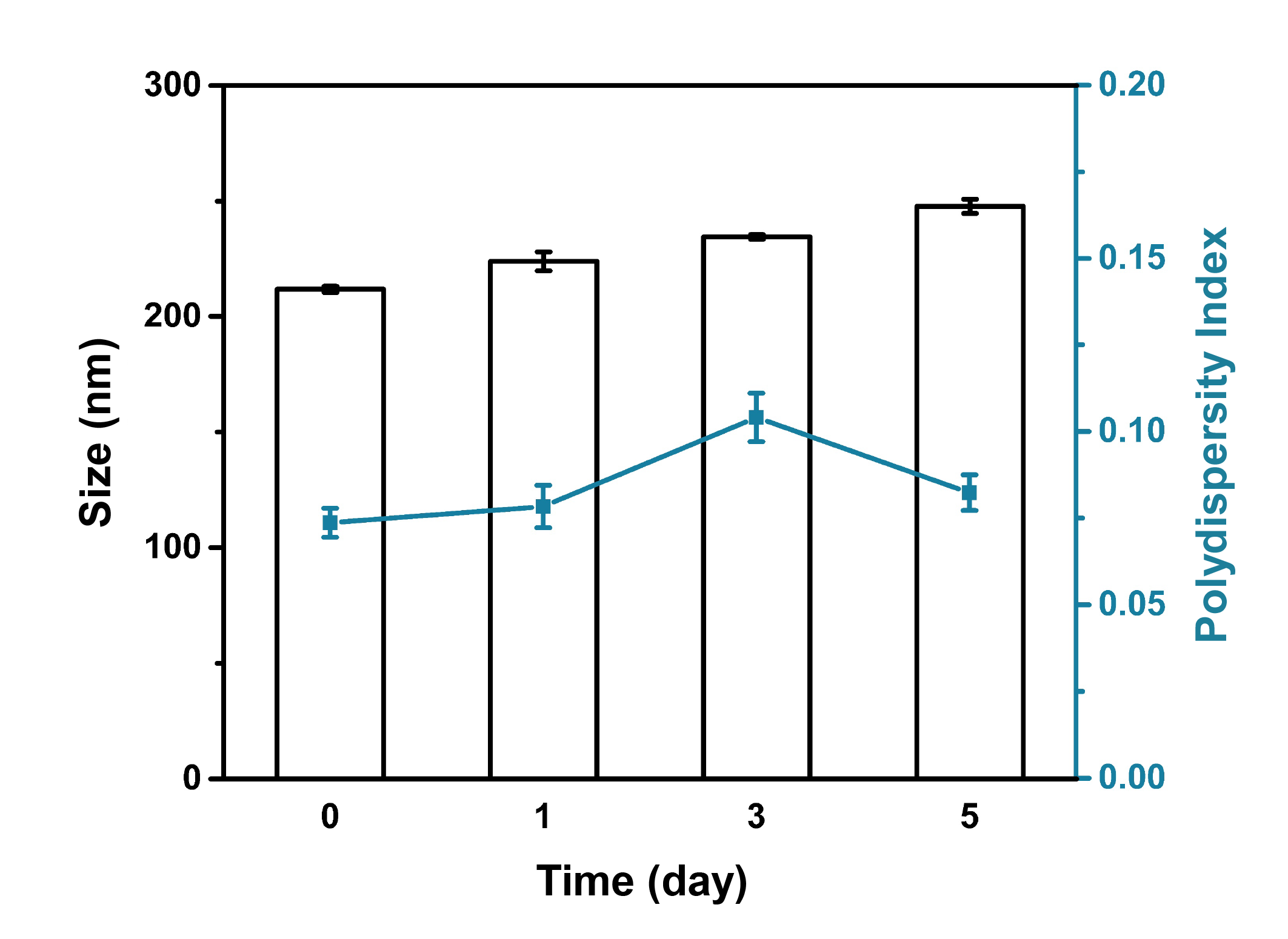
Supplementary Figure S1. The size and polydispersity index of MNP dissolved in serum-containing culture medium for different time.


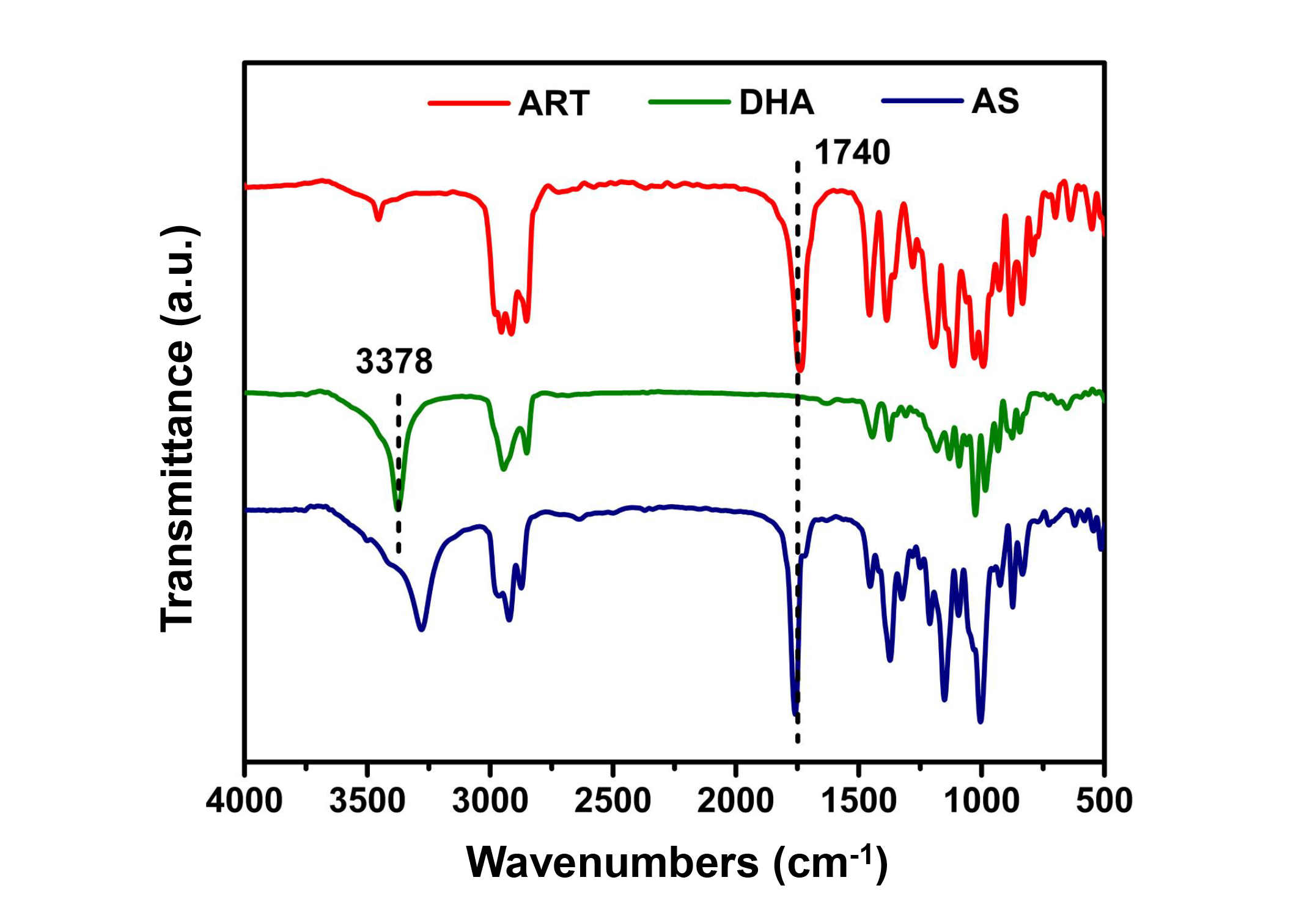


Supplementary Figure S2. FT-IR spectra of ART, DHA, and AS.


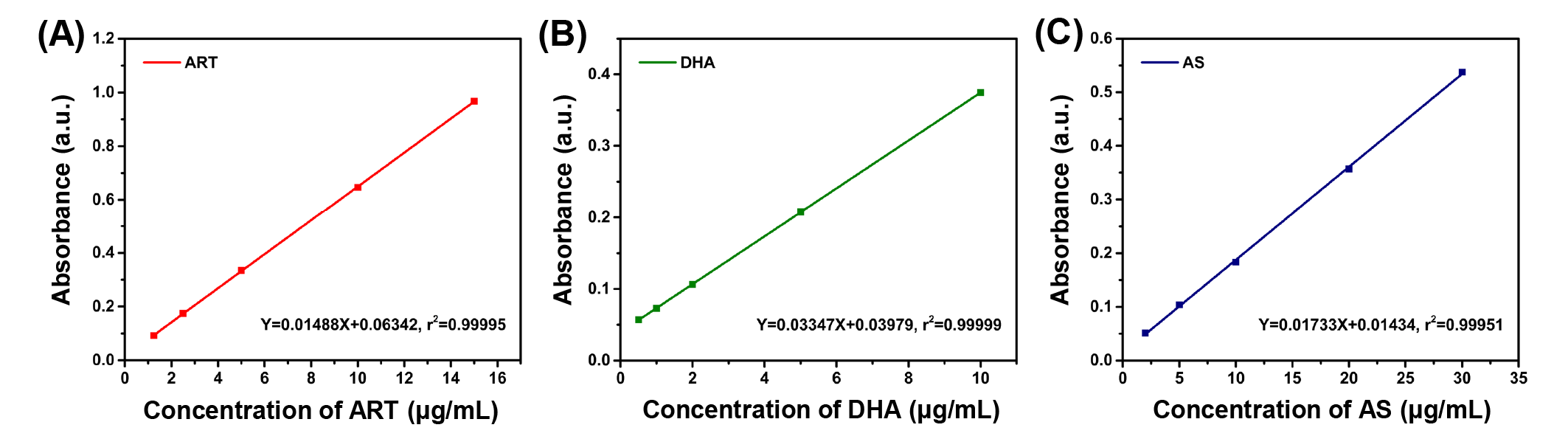


Supplementary Figure S3. Standard curves of (A) ART, (B) DHA, and (C) AS concentration.


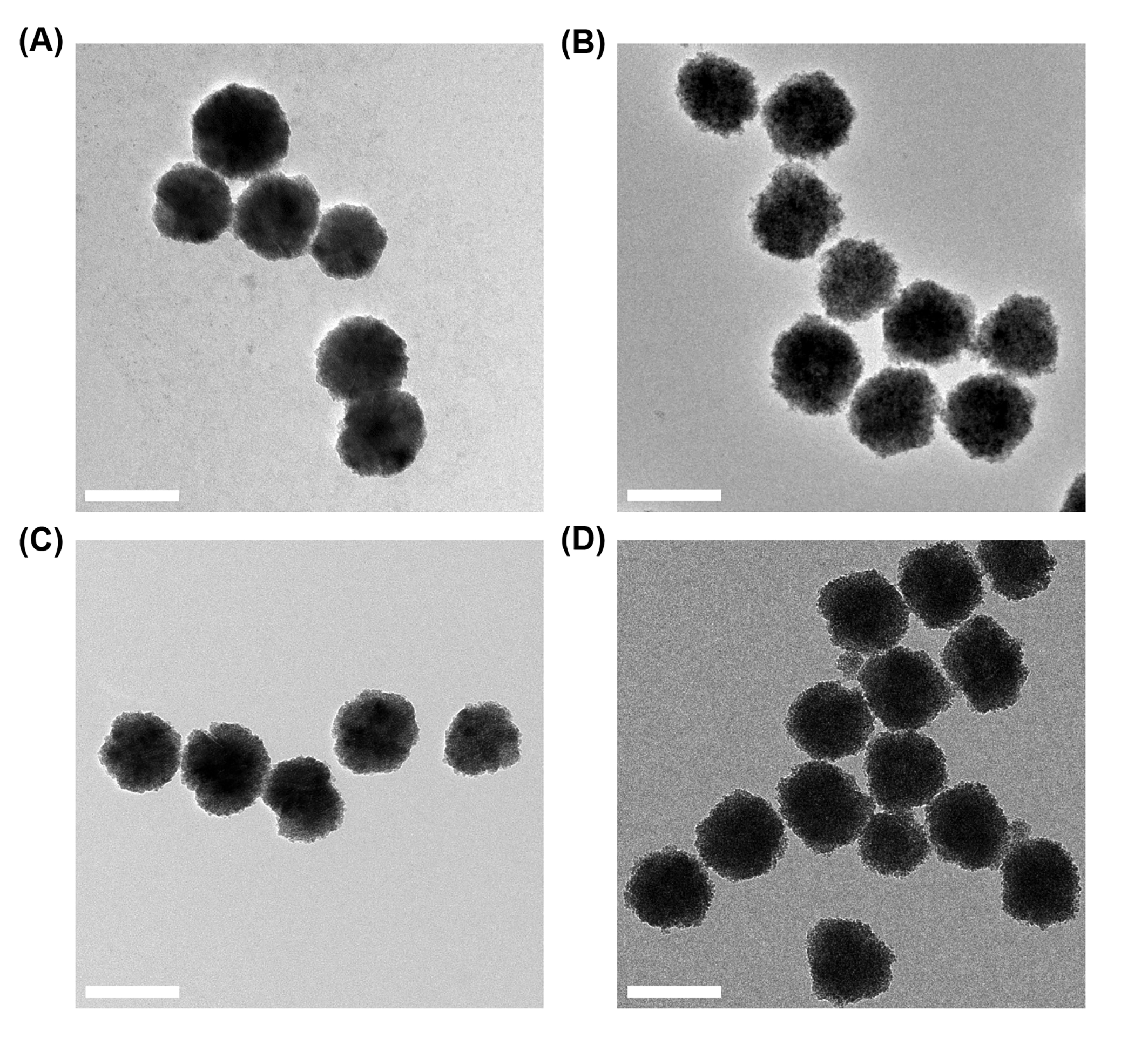


**Supplementary Figure S4.** TEM images of (A) blank MNP, (B) MNP-ART, (C) MNP-DHA and (D) MNP-AS. The scale bars represent 200 nm.


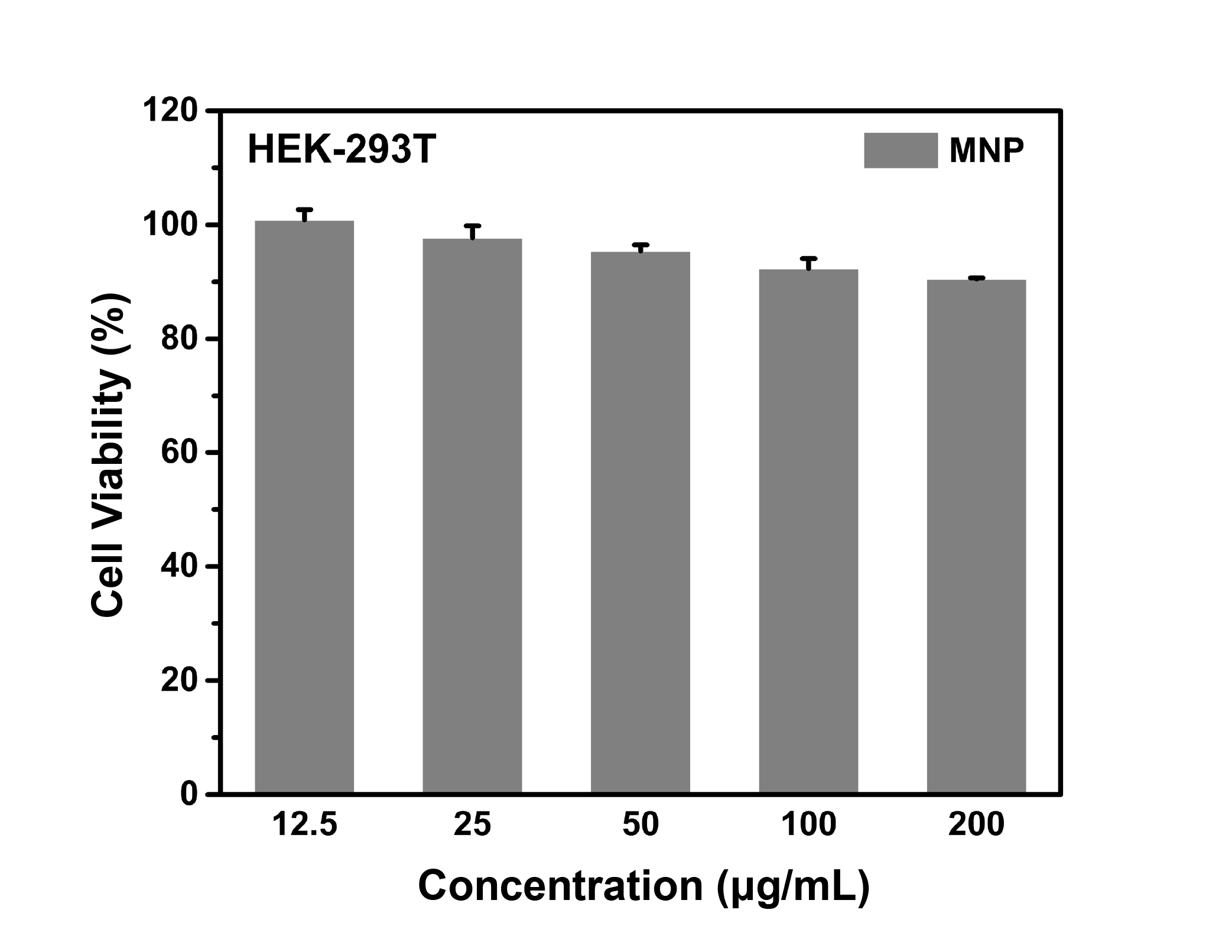


Supplementary Figure S5. Cell viability of HEK-293T cells after 24 h incubation with MNP suspensions at different concentrations.


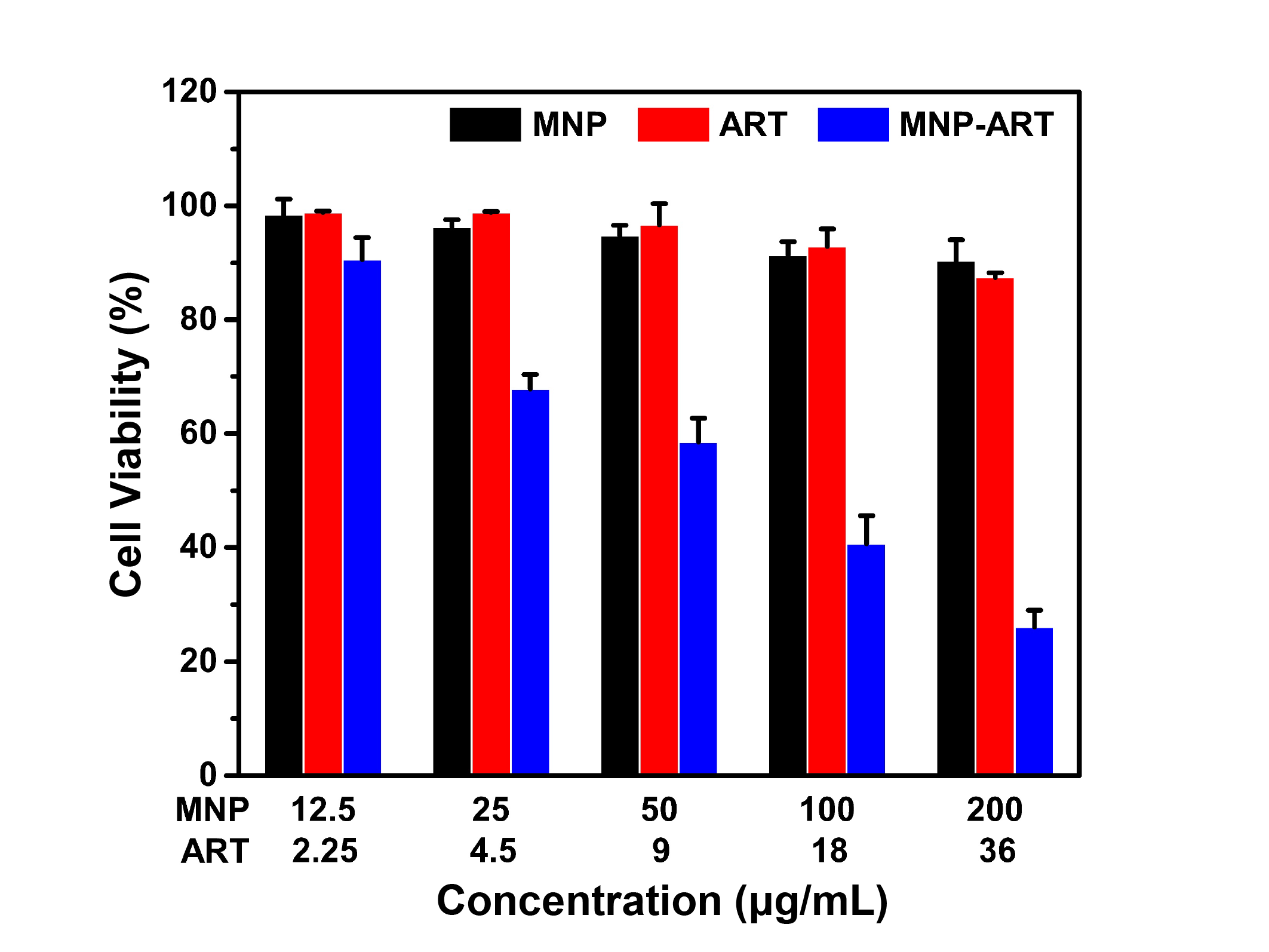


Supplementary Figure S6. Cell viability of MCF-7 cells after 48 h incubation with MNP, free ART, and MNP-ART suspensions at different concentrations.
